# True and Perceived Synchrony are Preferentially Associated With Particular Sensory Pairings

**DOI:** 10.1038/srep17467

**Published:** 2015-12-01

**Authors:** Jean-Paul Noel, Mark T. Wallace, Emily Orchard-Mills, David Alais, Erik Van der Burg

**Affiliations:** 1 Neuroscience Graduate Program, Vanderbilt University Medical Center, Nashville, TN 37240, USA; 2 Vanderbilt Brain Institute, Nashville, TN 37240, USA; 3 Department of Hearing and Speech Sciences, Vanderbilt University Medical Center, Nashville, TN 37240, USA; 4 Department of Psychology, Vanderbilt University, Nashville, TN 37240, USA; 5 Department of Psychiatry, Vanderbilt University, Nashville, TN 37240, USA; 6 Vanderbilt Kennedy Center, Nashville, TN 37240, USA; 7 School of Psychology, University of Sydney, Sydney, Australia; 8 Department of Cognitive Psychology, Vrije Universiteit Amsterdam, The Netherlands

## Abstract

Perception and behavior are fundamentally shaped by the integration of different sensory modalities into unique multisensory representations, a process governed by spatio-temporal correspondence. Prior work has characterized temporal perception using the point in time at which subjects are most likely to judge multisensory stimuli to be simultaneous (PSS) and the temporal binding window (TBW) over which participants are likely to do so. Here we examine the relationship between the PSS and the TBW within and between individuals, and within and between three sensory combinations: audiovisual, audiotactile and visuotactile. We demonstrate that TBWs correlate within individuals and across multisensory pairings, but PSSs do not. Further, we reveal that while the audiotactile and audiovisual pairings show tightly related TBWs, they also exhibit a differential relationship with respect to true and perceived multisensory synchrony. Thus, audiotactile and audiovisual temporal processing share mechanistic features yet are respectively functionally linked to objective and subjective synchrony.

## 

Among other factors, the spatial^1^,^2^,^3^ and temporal^3^,^4^,^5^ features of the stimuli in our environment play a critical role in the integration and binding of information across the different senses. Thus, in studies ranging from neurophysiological analyses of the activity pattern of individual neurons in animal models to studies of human perception and performance, the spatiotemporal relationships between multisensory stimuli have been shown to be important determinants in whether and how these cues are integrated and bound in order to shape behavior and perception^6^,^7^,^8^,^9^. On the human side, psychophysical paradigms have characterized various aspects of multisensory temporal function through measures such as the point of subjective simultaneity (PSS; point in time when stimuli from different senses are most likely judged to have occurred simultaneously) and the temporal binding window (TBW; epoch of time over which stimuli from different senses are bound together to alter behavior and perception)^10^,^11^,^12^,^13^,^14^,^15^.

It must be noted that the TBW is not a direct measure of multisensory integration, but rather indexes the likelihood with which individuals will categorized two asynchronously presented multisensory events as co-occurring in time, and has been demonstrated to reliably predict the temporal constraints with which participants integrate information across multiple senses^16^. In addition, the PSS reveals that subjective simultaneity rarely maps on to objective simultaneity (i.e., it is rarely 0, a likely result of the differences in propagation and transduction times for different sensory channels, thus reflecting the sensory statistics of the world), and the TBW reveals that multisensory stimuli are likely to be judged as co-occurring in time over fairly broad temporal windows (i.e., these are frequently on the order of several hundreds of milliseconds)^17^. A number of studies have demonstrated that stimuli from the different sensory modalities have the capacity to influence one another’s processing over long temporal intervals [for review see]^17^,^18^. Despite the utility and ubiquity of the PSS and TBW as tools to index various facets of multisensory temporal function^17^,^18^,^19^,^20^, the relationships between these measures across individuals and sensory combinations have not been systematically studied. Such an analysis has the potential to not only reveal mechanistic commonalities (as well as differences) between these indices, but also the ability to better elucidate whether certain sensory combinations and their binding is better rooted in objective versus subjective time. That is, it is conceivable that the distribution with which participants indicate sensory events as co-occurring in time may exhibit particular patterns across different sensory pairings. Audio-tactile and visuo-tactile events occur on the body of the observer, and thus, the differences in propagation times between these sensory modalities may have little impact on simultaneity judgments. Thus, it seems quite conceivable that audio-tactile and visuo-tactile pairings most commonly draw from a distribution of PSSs centered around SOA = 0 (objective synchrony). On the other hand, most audio-visual events occur at a variety of distances from the observer, thus necessitating the coding of different propagation asynchronies, and hence perhaps more faithfully reflecting that statistics of the sensory world (i.e., light does travel faster than sound). An analysis of whether particular multisensory pairings (e.g., audio-visual, audio-tactile, visuo-tactile) are more or less rooted in objective versus subjective timing is not only informative in that it can point toward a given pairing’s propensity to be influenced by recent experience, but also serves to reinforce that different sensory systems and their pairings may be more strongly yoked to the physical (i.e., objective timing) aspects of stimuli in the world whereas others may be more malleable based on recent (and past) sensory experiences.

In the current study, our goals were two-fold and were achieved by examining multisensory temporal function within and across participants using audio-tactile (AT), audio-visual (AV), and visuo-tactile (VT) simultaneity judgments (SJs). The SJ task is a simple task in which participants are asked to judge the simultaneity or asynchrony of a multisensory stimulus pairing presented in close temporal correspondence. In this task, one can derive both a PSS and TBW based on point and distributional measures. Our first goal was to relate PSS and TBW measures within individuals for the different sensory pairings. Our second goal was to relate these measures to real (i.e., objective) versus perceived (i.e., subjective) synchrony. That is, we attempt to establish a relationship, on a subject-by-subject basis, between TBW size and PSS, across different multisensory pairings, and across true synchrony (objective synchrony) and the statistically most encountered asynchrony (subjective simultaneity). Whereas objective synchrony is simply true physical simultaneity (i.e., SOA = 0 ms), subjective synchrony is operationally defined as the mean SOA at which a population of individuals is most likely to categorized the stimuli as occurring simultaneously. Thus, a particular individual’s PSS is a measure of the distance between true synchrony and that subject’s perception of synchrony, while the deviation of that particular subject’s PPS and the mean PPS of the whole sample (which is operationally taken to reflect statistical synchrony) is a measure of the distance between that subject’s perception of synchrony and subjective synchrony.

## Results

The current study sought to determine whether and how the point of subjective simultaneity (PSS) for multisensory stimuli, and the temporal binding window (TBW) for binding them, vary across participants and sensory pairings. Our goals were to reveal how TBWs relate to both true synchrony (i.e., physically aligned stimuli) and subjective synchrony (i.e., the perception of synchrony when stimuli are temporally offset to negate differential processing delays), and to reveal how prior exposure can influence the PSS and TBW.

The dataset consisted of simultaneity judgments (SJs) from eighteen subjects in three counterbalanced experiments using audio-visual (AV), audio-tactile (AT) and visuo-tactile (VT) stimuli. These data, which represent one of the few within-subjects account of SJ reports in the three dominant exteroceptive sensory pairings (AV, AT, and VT), were first collected for a published report examining the time scales of multisensory temporal recalibration [see]^20^. Although prior work has characterized simultaneity^21^,^22^ and temporal order^22^,^23^ judgments across these three modality pairings, those studies did not examine the relationships between PSS and TBW measures across and within the different modality pairings. Characterizing these relationships is vital to inferring the ecological contributions of different multisensory pairs in building a veridical and unified perception of the surrounding world.

### True and Perceived Synchrony Across Sensory Pairings

An individual’s PSS and TBW were defined as the mean (PSS) and standard deviation (TBW) of a Gaussian function fitted to their reports of synchrony as a function of SOA. In these plots, for audio-visual pairs (blue) the audio leading conditions is designed by negative values and the visual leading condition is designed by positive values. For the audio-tactile (red) and visuo-tacile (black) pairs, tactile leading is designed by positive values. Gaussian fittings for the measured distributions were highly accurate (all R^2^ > 0.96).

**Figure 1:**
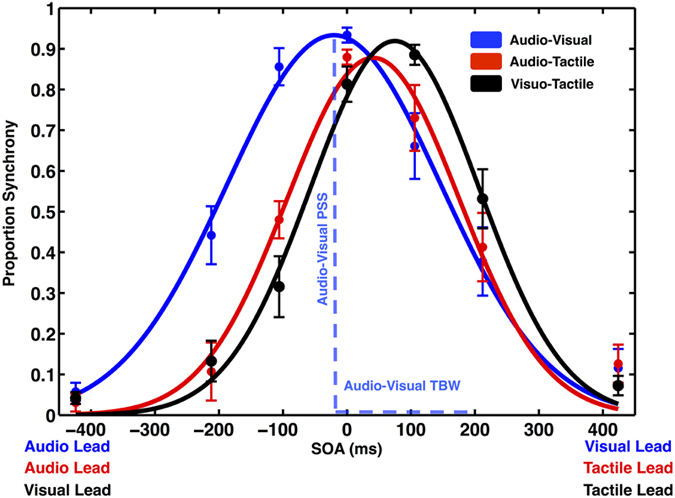
Simultaneity judgments for audio-visual, audio-tactile, and visuo-tactile pairs. Proportion of synchrony reports are plotted as a function of Stimulus Onset Asynchrony (SAO) and stimulus pair, audio-visual (blue), audio-tactile (red), and visuo-tactile (black). Gaussian curves are fitted to raw data and represented as solid lines. Point of subjective simultaneity (PSS) and temporal binding window (TBW) are represented by the distribution’s mean and standard deviation parameters, respectively, as illustrated for the audio-visual pair. Error bars represent ± 1 SEM.

As shown in Fig. 1, a within-subjects one-way ANOVA revealed that the mean *distance* between true and perceived simultaneity (i.e., distance between SOA = 0 and SOA = PSS) across the AV, AT, and VT pairs were significantly different (F (2, 34) = 89.26, p < 0.001, partial η^2^ = 0.84). Whereas the perceived synchrony of VT pairings was furthest in time from objective simultaneity (M = 73.41 ms, S.E.M = 5.25 ms), the mean PSS for AT pairs was at an intermediate distance (M = 51.71 ms, S.E.M = 9.25 ms), and the mean PSS for AV pairings was judged closest to (and not statistically different from) objective synchrony (M = 9.06 ms, S.E.M = 9.34 ms; p = 0.34). Comparisons across all combinations of sensory pairs (AV-AT, AV-VT, and AT-VT) revealed them to be statistically different from each other (AV-AT p < 0.001, AV-VT p < 0.001, AT-VT p = 0.014). To the best of our knowledge, this relationship (| AVpss | < |ATpss| < |VTpss|) has not been previously reported in the literature and points to a different relationship between true and perceived synchrony across the different sensory pairings. It must be noted, however, that as previously mentioned, PSS-values are known to vary as a function of stimuli characteristics, and thus one must be cautious of drawing conclusions based on this single measure (i.e., PSS).

### TBW Across Sensory Modalities

As illustrated in Fig. 1, the width of the TBW differed for the various modality pairings (F (2, 34) = 15.12, p < 0.001, partial η^2^ = 0.47). The AV pair displayed the largest temporal binding window (M = 175.80, S.E.M = 21.15), which was significantly greater than that of the AT (M = 139.17, S.E.M = 16.62) and VT (136.04, S.E.M = 14.71) pairs (Bonferroni-corrected p < 0.001 and p < 0.01, respectively). The TBW for the AT and VT pairs did not differ from each other (p = 0.66). Our findings (AV_tbw_ > AT_tbw_ , AV_tbw_ > VT_tbw_, and AT_tbw_ = VT_tbw_) are in partial agreement with a previous account^22^ indicating that the TBW for AV pairings is larger than for other multisensory pairs. However, our work differs somewhat from this previous study^22^, as we did not find a significant difference between the AT and VT pairs. Regardless, the emergent picture from both studies (the current and)^22^ is that seemingly those multisensory pairings with a tactile component exhibit the smallest TBWs, an observation likely underpinned by the temporal properties of the somatosensory system being the most reliable and impermeable to outside factors^24^.

The fact that different multisensory pairings demonstrate unique PSSs and TBWs indicates that the temporal characteristics of multisensory integration are dependent upon the modalities that are paired, in addition to stimuli features^19^,^25^. This makes good ecological sense, as the different sensory energies travel from their source at different speeds and the different sensorineural systems have distinct transduction and neural propagation times^26^,^27^,^28^ for multisensory latencies review]. Furthermore, previous work has demonstrated that the PSS and TBW are dependent upon stimulus features and on task contingencies, with the window widening as stimulus complexity increases^19^,^20^. Despite this, prior work has failed to examine how these measures relate to one another across multisensory pairings and within a given individual. Such an analysis may reveal commonalities in the neural architecture supporting multisensory temporal function and provide insights into how multisensory temporal relations are differentially weighted in the internal representation of our sensory world.

### TBW Correlates Across Modality Pairings Within Individuals

**Figure 2:**
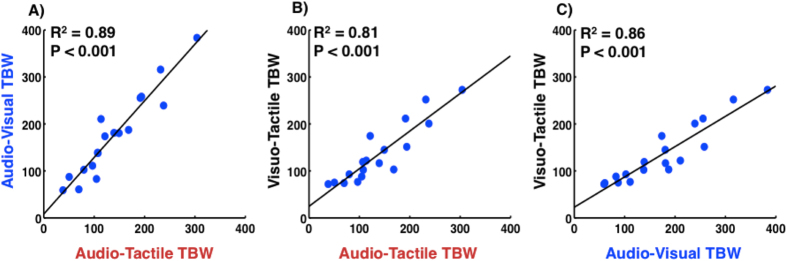
Relationship between TBW widths of different modality pairings. (**A**) Width of the audio-visual TBW as a function of the width of the audio-tactile TBW. (**B**) Width of the visuo-tactile TBW as a function of the width of the audio-tactile TBW. (**C**) Width of the visuo-tactile TBW as a function of the width of the audio-visual TBW. Solid black lines through each dot cluster represents the linear regression for that pairing.

Pearson’s r correlations were calculated between the widths of the TBW for all combinations of sensory pairs (AV-AT, VT-AT, and VT-AV). As depicted in Fig. 2, all three associations revealed a high correlation (AV-AT R^2^ = 0.89, p < 0.001; VT-AV R^2^ = 0.86, p < 0.001; VT-AT R^2^ = 0.81, p < 0.001).

To examine these relationships further and rule out possible confounding influences related to cognitive and decisional biases, the data were subject to signal detection theory (SDT) analysis. For each participant and for each sensory combination, the 90 synchronous trials (SOA = 0) were compared with another 90 trials drawn randomly from the set of 540 asynchronous trials (those with SOAs =  ± 420, ± 120, ± 60 ms). We then applied SDT analysis by calculating sensitivity (i.e., d′) and response bias (i.e., criterion *c*) for that sample. This was performed iteratively 1000 times, each time re-sampling a random selection of 90 asynchronous trials, to build a distribution of d′- and c-values for each participant in each sensory pairing. The distributions were then averaged and subjected to correlational analyses (see Supplementary information online for details). Results indicated that response bias correlated significantly for VT-AT SJs (R^2^ = 0.45, p = 0.04), showed a trend for AV-AT (R^2^ = 0.40, p = 0.06), and did not correlate for the VT-AV pair (R^2^ = 0.04, p = 0.58) (See Supplementary Fig S1). Importantly, none of these correlations explained as much variance as did the correlations between the different TBWs. Partial correlational analyses accounting for the variance explained by cognitive factors demonstrated significant relations between the widths of the TBWs (all R^2^s > 0.48, all ps < 0.02). This analysis demonstrates that while response biases certainly contributed to the correlations, cognitive factors were not the sole driving force in the relationships between the different TBWs.

The existence of strong correlations between TBWs for all combinations of sensory pairs in individual participants suggests that these temporal windows vary among individuals in a consistent manner. If this were truly the case, the width of a particular TBW may also relate to other multisensory temporal processing parameters, such as the PSS or the point of true synchrony (SOA = 0 ms).

The PSS represents the temporal offset between two stimuli in different senses at which subjects are most likely to report synchrony. Whereas objective synchrony is a stimulus onset asynchrony of zero, the PSS rarely matches true synchrony^17^,^18^,^19^,^20^. The current dataset provides a unique opportunity to compare whether different sensory pairings preferentially associate with true (objective) or perceived (subjective) synchrony. The TBWs of these different sensory pairings, represented by the widths (e.g., standard deviations) of their synchrony distributions, should vary as a function of distance between the distribution’s peak and the type of synchrony it is preferentially associated with. That is, if a particular sensory pairing were primarily representing or associated with true synchrony, the TBW should increase as the distribution’s peak moves further away from zero. On the other hand, if a sensory pairing were encoding subjective synchrony (namely, maintaining a moving average of the sensory asynchronies it encounters in the world) the TBW should not co-vary with distance from true simultaneity, but rather with distance from subjective simultaneity, that is, the sample’s mean PSS. This holds from the fact that as representation become inaccurate (i.e., not representing what they ought), they conceivably may also become less precise and thus trade-off accuracy and precision.

### AT and AV Pairs Differentially Encode For True And Subjective Synchrony

**Figure 3:**
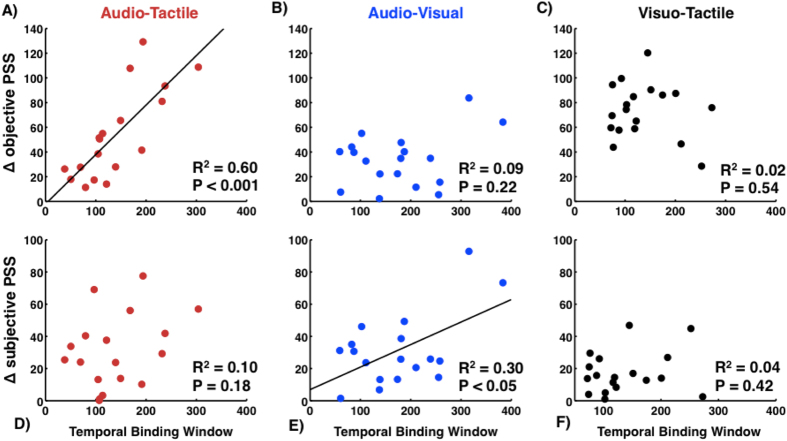
Relationship between TBW widths for different modality pairings and distance from true and perceived simultaneity. Distance between the peak of simultaneity reports for individual participants and true synchrony (SOA = 0) is significantly correlated for the audio-tactile pair **(A**), but not the audio-visual (**B**) or visuo-tactile (**C**) pairs. Conversely, the distance between an individual’s PSS and mean perceived synchrony are correlated for the audio-visual (**E**) but not the audio-tactile (**D**) or visuo-tactile (**F**) pairs.

To test the relationship between the size of the TBW and objective synchrony, we simply correlated the TBW with the absolute distance of the distribution’s peak from zero. To test the relation between TBW width and subjective synchrony, we calculated the distance between each participant’s PSS and the group’s mean PSS and correlated this with TBW size. As depicted in Fig. 3 (upper row), the size of the AT TBW was positively correlated with objective simultaneity (SOA = 0), (R^2^ = 0.60, p < 0.001). Thus, as the delta relative to true simultaneity increased, the width of the TBW increased. In contrast, no relationship was found between the distance from objective synchrony and TBW for the AV (R^2^ = 0.0 9, p = 0.22) or VT (R^2^ = 0.02, p = 0.54) pairs. On the other hand, when plotted in terms of distance to subjective synchrony, TBW increased significantly for the AV pair (R^2^ = 0.30, p < 0.05, Fig. 3, lower row) but no correlation was found for the AT (R^2^ = 0.10, p = 0.18) or VT (R^2^ = 0.04, p = 0.42) pairs.

These data suggest that whereas the temporal binding of AT stimuli is primarily associated with true synchrony, AV binding is more strongly reflecting the perceived stimulus timing. Stated differently, AV timing is seemingly most strongly associated with a running average of the statistical synchrony of the world. This finding fits nicely within the existing literature indicating a superior synchrony resolution for AT stimulus pairs, as compared to AV and VT pairs^22^, and with evidence for faster^21^ and more flexible^23^ recalibration to novel temporal relations of AV pairs over AT and VT pairs.

### PSSs Do Not Correlate Across Multisensory Pairings

Lastly, for all combinations of sensory pairings, we tested whether PSS values for one sensory pair correlated with PSSs in any of the other pairs. We did this separately for individual PSS distance from group mean PSS, and for individual PSS distance from objective synchrony. If encoding for objective synchrony were largely environment-independent, we would expect that the variance within a population would result from intrinsic properties and therefore be maintained across sensory and multisensory pairings. The same argument holds for the processing of subjective synchrony. If, on the other hand, perceived synchrony were determined by the statistics of the environment and prior multisensory experiences, we would expect no correlations between PSSs.

**Figure 4:**
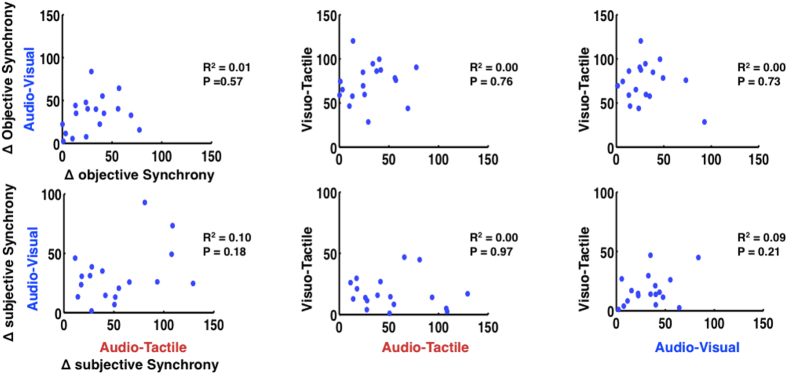
Relationship between PSS and true and perceived simultaneity as a function of multisensory pairings. Neither objective (top row) nor subjective (bottom row) distance from synchrony was correlated among individuals across multisensory pairings.

As shown in Fig. 4, none of the combinations of multisensory pairings (AV-AT, VT-AT, and VT-AV) revealed a correlation with distance from objective (true) or subjective (statistical) synchrony (all p > 0.18). These results suggest there is no systematic mapping between a particular individual’s PSS across multiple combinations of sensory modalities. When compared with the strong TBW correlations between multisensory pairings (Fig. 2), which suggest that TBWs are more strongly associated with intrinsic processing characteristics and thus more fixed representational processes, the PSS appears to be more labile and more strongly linked to environmental statistics. Indeed, although recent work has focused on the malleability of the TBW, earlier studies have highlighted the highly plastic nature of the PSS^29^,^30^.

## Discussion

The results of the current study demonstrate that temporal binding of sensory signals from different modalities is strongly determined by intrinsic factors likely involving shared neural resources. The strong correlations between TBW widths across all three combinations of multisensory pairings (AV-AT, VT-AT, and VT-AV) indicate that the degree to which an individual binds multisensory information is highly individualistic but strongly yoked across the sensory systems. This stems from the fact that each individual is likely exposed to distinct temporal relations as they sample the statistical relationships of objects and events in their sensory world. In stark contrast, the absence of correlations between PSSs across the different sensory pairings suggests this measure to be more malleable and thus dependent upon the changing sensory statistics of the world (rather than being strongly shaped by individual factors). This suggestion is consistent with existing literature indicating extremely rapid recalibration of PSS (e.g., on a trial-by-trial basis^21^), whereas the TBW has only been reported to change after extensive exposure to and training with new temporal relations^10^,^23^.

Multisensory TBWs, in addition to being closely related within individuals regardless of sensory combination, appear to play several distinct functional roles. The size of an individual’s AT TBW correlates positively with the distance between their PSS and true synchrony (i.e., an SOA = 0). That is, the width of the AT TBW behaves as if grounded in true synchrony, a feature not found in either the AV or VT TBW. On the other hand, the width of the AV TBW (and not AT or VT TBW widths) increased as a function of the distance between a particular subject’s PSS and ‘statistical synchrony’, defined as the mean PSS of the sample tested. This relationship suggests that the AV TBW behaves as if rooted in subjective synchrony. Certainly, external events are converted into neural/perceptual ones in order to enable the nervous system to have access to them, and thus, temporal processing across sensory modalities are unlikely to be precisely and entirely grounded in objective/subjective synchrony. However, a large proportion of audio-tactile and visuo-tactile events are self-generated and happen within one’s peri-personal space, thus reducing the impact of differential propagation times and providing for a ground truth for physical synchrony that is continually reinforced as we interact with the world around us. On the other hand, most audio-visual events happen at a (variable) distance from observer, thus reflecting most veridically the sensory statistics of the world (i.e., propagation times).

The suggestion that the AT TBW behaves as if anchored in true synchrony while the AV TBW appears as if anchored in perceived synchrony is consistent with the fact that the AT pair is the most stable (as AT – as well as VT – events have consistent temporal relations since by definition they happen on the body and hence in a restricted spatiotemporal registry) and possesses the highest temporal resolution [see^22^ for demonstration of higher temporal resolution for AT over AV and VT]^24^. In contrast, the AV pair has routinely been shown to be the most malleable^23^ and the fastest^21^ to recalibrate to new temporal relationships. In addition, AV pairings are arguably more common in human behavior (and exhibit the widest range of spatial locations), and thus may ‘necessitate’ of a more flexible recalibration system. Collectively, these findings are highly consistent with the modality appropriateness hypothesis^31^,^32^, which states that the reliability of information from the individual modalities determines their relative weighting when forming a multisensory percept.

Finally, although the width of the VT TBW correlates with the width of the AV and AT TBWs – implying shared mechanisms – a functional role for this modality pairing was not identified, as its width did not show a systematic relationship with any other multisensory temporal property. In speculating on this lack of association, it might be that whereas the AT and AV multisensory pairs respectively code for temporal aspects of the stimulus pairs (i.e., true and perceived synchrony), the VT pair is most closely associated with spatial, rather than temporal aspects of the multisensory pairing. This speculation is consistent with the fact that both visual and tactile representations are highly spatiotopic (as opposed to auditory representations which tend to be strongly frequency or time selective), and that peri-personal and extra-personal space boundaries are most strongly delineated by bimodal visuo-tactile neurons^33^,^34^. In addition to exploring the relative contributions of VT pairs to the assembly of multisensory spatial representations, future work needs to delve more deeply into the spatiotemporal characteristics of multisensory function, given that space and time are inextricably linked in the statistics of stimuli in our world.

## Materials and Methods

Eighteen participants (nine females; mean age 23.7 years, range 19–39) took part in three separate experiments; an audio-visual, an audio-tactile, and a visuo-tactile simultaneity judgment task (SJ-task). All subjects reported normal vision, hearing, and touch. Written informed consent was obtained from all participants, and all experiments were approved by the local ethics committee of the University of Sydney and adhered to the tenets of the Declaration of Helsinki. Stimuli and apparatus utilized have been previously reported^21^, and are therefore only briefly described here. Visual stimulus was a white ring (radius 2.6°; width 0.4°), presented around a white fixation dot on a black background (<0.5 cd/m^2^) for a duration of 50 ms. The auditory stimulus was a pure tone (500 Hz; 50 ms duration, sampling rate 44 100 Hz). Visual stimulus was presented on a CRT monitor (Sony CPD-E400; resolution 1 280 × 1 240; refresh rate 85 Hz), while the auditory one was presented using headphones (Sennheiser HD 380 pro) at a comfortable suprathreshold listening level. The tactile stimulus was a salient, pure sine wave (50 Hz; 50 ms duration; sampling rate of 44 100 Hz), presented using an exposed speaker (Edifier M1250) which was mounted on a foam block. Participants held this block and placed the index finger of their dominant hand on the exposed drum. Testing was carried out in a dimly lit room, and utilizing E-Prime 2.0 software. Regardless of the multisensory pairing being tested, each trial began with a white fixation cross on a black screen for 800 ms. Subsequently a stimulus in one of the modalities being tested was presented, and at a given stimulus onset asynchrony (SOA), the stimuli in the second sensory modality was presented. The SOAs varied across trials between ± 424, ± 212, ± 106, and 0ms (true synchrony). Negative and positive values indicating that either of the two sensory modalities being tested could precede the other. Participants were required to judge whether the two stimuli were presented simultaneously or not. Each experiment consisted of 630 trials (i.e., 90 trials per SOA). The SOAs were randomized and the order of experiments was counterbalanced across participants.

## Additional Information

**How to cite this article**: Noel, J.-P. *et al.* True and Perceived Synchrony are Preferentially Associated With Particular Sensory Pairings. *Sci. Rep.* **5**, 17467; doi: 10.1038/srep17467 (2015).

## Supplementary Material

Supplementary Information
