# Supplementary material for: True and Perceived Synchrony are Preferentially Associated With Particular Sensory Pairings
Source: Sci Rep. 2015 Dec 1;5:17467. doi: 10.1038/srep17467 (PMC4664927; doi:10.1038/srep17467)
Supplement: Supplementary Information [file srep17467-s1.doc]

**SUPPLEMENTARY INFORMATION TO;**

True and Perceived Synchrony

are Preferentially Associated

With Particular Sensory Pairings

Jean-Paul Noel^1, 2, *^, Mark T. Wallace^2, 3, 4, 5, 6, *^, Emily Orchard-Mills^7^, David Alais^7^, & Erik Van der Burg^7, 8^

^1^ Neuroscience Graduate Program, Vanderbilt University Medical Center, Nashville, TN 37240, USA

^2^ Vanderbilt Brain Institute, Nashville, TN 37240, USA

^3^ Department of Hearing and Speech Sciences, Vanderbilt University Medical Center, Nashville, TN 37240, USA

^4^ Department of Psychology, Vanderbilt University, Nashville, TN 37240, USA

^5^ Department of Psychiatry, Vanderbilt University, Nashville, TN 37240, USA

^6^ Vanderbilt Kennedy Center, Nashville, TN 37240, USA

^7^ School of Psychology, University of Sydney, Sydney, Australia

^8^ Department of Cognitive Psychology, VU University Amsterdam, The Netherlands

* Corresponding Authors:

Jean-Paul Noel Dr. Mark Wallace

[Jean-paul.noel@vanderbilt.edu](mailto:Jean-paul.noel@vanderbilt.edu) [mark.wallace@vanderbilt.edu](mailto:mark.wallace@vanderbilt.edu)

[+1] 615-936-7108 [+1] 615-936-7108

7110 MRB III Bio Sci Bldg 7110 MRB III Bio Sci Bldg

465 21^st^ Ave South 465 21^st^ Ave South

Nashville, TN 37232, USA Nashville, TN 37232, USA

Running Title: Modality-Pairing Temporal Binding Windows

Keywords: Multisensory, Simultaneity, Audio-Visual, Audio-Tactile, Subjectivity, Objectivity

To examine the relationship between the size of temporal binding windows (TBW) within participants and across multisensory pairings (audio-visual/audio-tactile, audio-visual/visuo-tactile, and visuo-tactile/audio-tactile), and in order to rule out possible confounding influences related to cognitive and decisional biases, the data were subject to signal detection theory (SDT) analysis. For each participant and for each sensory combination, the 90 synchronous trials (SOA = 0) were compared with another 90 trials drawn randomly from the set of 540 asynchronous trials (those with SOAs = ±420, ±120, ±60). We then applied SDT analysis by calculating sensitivity (i.e., d’) and response bias (i.e., criterion *c*) for that sample. This was performed iteratively 1000 times, each time re-sampling a random selection of 90 asynchronous trials, to build a distribution of d’- and c-values for each participant in each sensory pairing. The distributions were then averaged and subjected to correlational analyses. It is important to note that, these distributions can vary from one simulation to another, and thus reported d’- and c-values can vary as well. In addition, it is also important to note that when participants do not commit errors (i.e., not reporting synchronous when in fact stimuli were presented with an SOA = 0ms), signal detection theory is not feasible. Indeed, this was the case for half our sample (n = 9), and thus SDT analysis was only possible based on values obtained from 9 participants. Results emanating from this analysis should be cautiously interpreted (because of the small sample size and the non-deterministic nature of the simulations).

The average d’-values indicate that, in general, participants were accurate at detecting synchrony vs. asynchrony (audio-visual M = 1.61, S.E.M = 0.17; audio-tactile M = 1.58, S.E.M = 0.15; visuo-tactile M = 1.38, S.E.M = 0.14). These values did not differ significantly one from another (F(2, 16) = 2.110, p = 0.158), but were indeed significantly different from zero (One-Sample t-test to zero; all p < 0.001). Similarly, c-values did not differ among multisensory pairings (F(2, 16) = 3.574, p = 0.095), and in this case, were also not significantly different from zero (all p > 0.19), indicating that no substantial response bias existed.

In terms of c-values, as illustrated in Fig S1, results indicated that response bias correlated significantly for VT-AT SJs (R^2^ = 0.45, p = 0.04), showed a trend for AV-AT (R^2^ = 0.40, p = 0.06), and did not correlate for the VT-AV pair (R^2^ = 0.04, p = 0.58) (See Supplementary Fig S1). Importantly, none of these correlations explained as much variance as did the correlations between the different TBWs. Partial correlational analyses accounting for the variance explained by cognitive factors demonstrated significant relations between the widths of the TBWs (all R^2^s > 0.48, all ps < 0.02). This analysis demonstrates that while response biases certainly contributed to the correlations, cognitive factors were not the sole driving force in the relationships between the different TBWs.


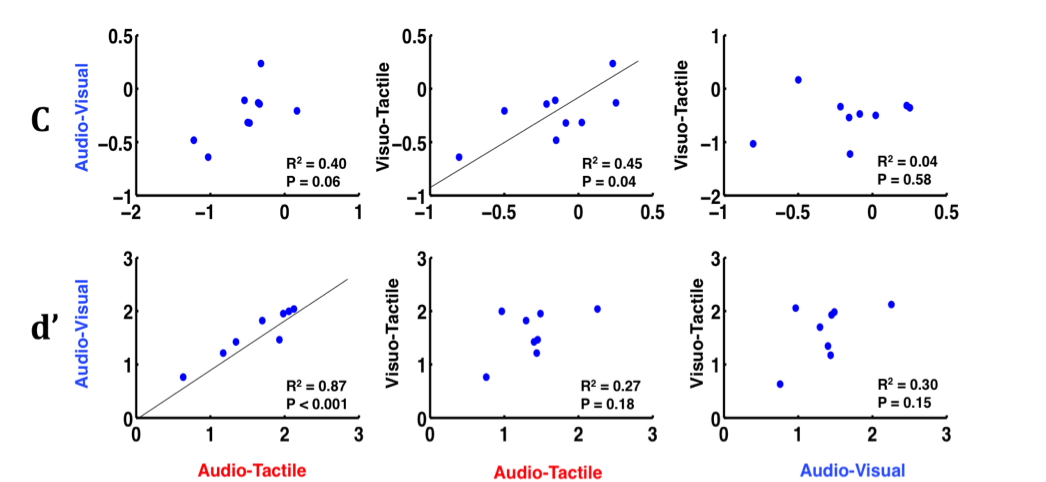


Figure S1. Correlation between response criterion (c) and sensitivity (d’) to synchronous vs. asynchronous stimulation as a function of multisensory pairings (audio-visual, audio-tactile, or visuo-tactile). Top panels represent the relationship between c-values, while lower panels indicate the relation between d’-values.

Lastly, with regard to the relationship between d’-values across multisensory pairings, results indicated that sensitivity to synchrony correlated significantly for the audio-visual and audio-tactile pairings (R^2^ = 0.87 P < 0.001), but not for the others (although they did show a trend, all p > 0.15). Thus, we conclude that these correlations are likely the result of a combination of cognitive and perceptual factors.
